# Combined Support Vector Machine Classifier and Brain Structural Network Features for the Individual Classification of Amnestic Mild Cognitive Impairment and Subjective Cognitive Decline Patients

**DOI:** 10.3389/fnagi.2021.687927

**Published:** 2021-07-30

**Authors:** Weijie Huang, Xuanyu Li, Xin Li, Guixia Kang, Ying Han, Ni Shu

**Affiliations:** ^1^State Key Laboratory of Cognitive Neuroscience and Learning, Beijing Normal University, Beijing, China; ^2^Center for Collaboration and Innovation in Brain and Learning Sciences, Beijing Normal University, Beijing, China; ^3^Beijing Key Laboratory of Brain Imaging and Connectomics, Beijing Normal University, Beijing, China; ^4^Department of Neurology, Xuanwu Hospital of Capital Medical University, Beijing, China; ^5^Department of Neurology, Beijing Friendship Hospital, Capital Medical University, Beijing, China; ^6^School of Electrical Engineering, Yanshan University, Qinhuangdao, China; ^7^Measurement Technology and Instrumentation Key Lab of Hebei Province, Qinhuangdao, China; ^8^Beijing University of Posts and Telecommunications, Beijing, China; ^9^Biomedical Engineering Institute, Hainan University, Haikou, China; ^10^Center of Alzheimer’s Disease, Beijing Institute for Brain Disorders, Beijing, China; ^11^National Clinical Research Center for Geriatric Disorders, Beijing, China

**Keywords:** subjective cognitive decline, mild cognitive impairment, support vector machine, white matter, diffusion tensor imaging

## Abstract

**Objective:**

Individuals with subjective cognitive decline (SCD) or amnestic mild cognitive impairment (aMCI) represent important targets for the early detection and intervention of Alzheimer’s disease (AD). In this study, we employed a multi-kernel support vector machine (SVM) to examine whether white matter (WM) structural networks can be used for screening SCD and aMCI.

**Methods:**

A total of 138 right-handed participants [51 normal controls (NC), 36 SCD, 51 aMCI] underwent MRI brain scans. For each participant, three types of WM networks with different edge weights were constructed with diffusion MRI data: fiber number-weighted networks, mean fractional anisotropy-weighted networks, and mean diffusivity (MD)-weighted networks. By employing a multiple-kernel SVM, we seek to integrate information from three weighted networks to improve classification performance. The accuracy of classification between each pair of groups was evaluated via leave-one-out cross-validation.

**Results:**

For the discrimination between SCD and NC, an area under the curve (AUC) value of 0.89 was obtained, with an accuracy of 83.9%. Further analysis revealed that the methods using three types of WM networks outperformed other methods using single WM network. Moreover, we found that most of discriminative features were from MD-weighted networks, which distributed among frontal lobes. Similar classification performance was also reported in the differentiation between subjects with aMCI and NCs (accuracy = 83.3%). Between SCD and aMCI, an AUC value of 0.72 was obtained, with an accuracy of 72.4%, sensitivity of 74.5% and specificity of 69.4%. The highest accuracy was achieved with features only selected from MD-weighted networks.

**Conclusion:**

White matter structural network features help machine learning algorithms accurately identify individuals with SCD and aMCI from NCs. Our findings have significant implications for the development of potential brain imaging markers for the early detection of AD.

## Introduction

Subjective cognitive decline (SCD) refers to self-perceived cognitive decline relative to a previously normal status, without impaired performance on standardized neuropsychological tests (Jessen et al., 2014; Molinuevo et al., 2017). There is gathering evidence that SCD may be the first symptomatic manifestation of Alzheimer’s disease (AD) occurring prior to amnestic mild cognitive impairment (aMCI) (Jessen et al., 2014; Rabin et al., 2017). Patients with aMCI, even those who temporarily revert to normal cognition, are at higher risk of progressing to dementia than age-matched normal controls (NCs) (Petersen et al., 2018). Effective intervention to delay or prevent pathologic cognitive decline may best be targeted at the SCD or MCI stage, in which cognitive function is still relatively preserved (Smart et al., 2017; Petersen et al., 2018). In consideration of this, it is critical to find sensitive, low-cost methods for the early detection of individuals at risk for further cognitive decline and incident AD dementia.

Recent advances in neuroimaging research suggest that elderly people with SCD have an increased likelihood of AD biomarkers across a range of modalities (Rabin et al., 2017). Diffusion tensor imaging (DTI) is a quantitative MRI technique that has been applied to delineate white matter (WM) microstructure through the characterization of the underlying water molecule diffusion (Amlien and Fjell, 2014). Using DTI measures, previous studies observed WM abnormalities in SCD subjects compared with the normal control (NC) group (Selnes et al., 2012; Li et al., 2016). Such alterations may predict medial temporal lobe atrophy and dementia (Selnes et al., 2013). In addition to the raw features obtained from DTI, characterization of the global architecture or topological property of WM connection patterns has recently drawn a great deal of interest (Sporns et al., 2005; Bullmore and Sporns, 2009). Previous studies suggested that patients with SCD and MCI exhibit global disruption of brain connectivity and topologic alterations of the whole-brain connectome rather than in a single isolated region (Shu et al., 2012, 2018). The topographical metrics of patients with SCD and MCI correlating with impaired cognitive performance suggest their potential use as biomarkers for the early detection of cognitive impairment in elderly individuals.

Over the past decades, neuroimaging measures have been increasingly integrated into imaging signatures of AD by means of classification frameworks, offering promising tools for individualized diagnosis and prognosis (Sajda, 2006; Rathore et al., 2017). Peter et al. (2014) suggested that, even at the SCD stage, structural MRI combined with the SVM method is a sensitive method for identifying subtle brain changes that correspond to future memory decline. Although SVM has been used successfully in several AD and MCI imaging studies involving WM connectivity network measure-based methods (Wee et al., 2012; Prasad et al., 2015; Rathore et al., 2017), it is scarce in SCD populations.

In this study, we wanted to assess the usefulness of multiple-kernel SVM approaches to accurately identify SCD and aMCI patients from normal aging based on different weighted structure networks. The primary aim of this study was to combine multiple weighted networks using multiple-kernel SVM with an SVM machine learning algorithm for each single weighted structure network approach and the direct data fusion method. The study further investigated the effect of feature number and constraint parameter C on classifying NC, SCD, and aMCI. Finally, information on which regions contributed most to the group separation was assessed, allowing for different types of discriminative features to be interpreted with respect to the underlying neurobiology of SCD and aMCI.

## Materials and Methods

### Subjects

This study included 138 right-handed and Mandarin-speaking subjects (51 NC, 36 SCD, and 51 aMCI) who were recruited at the memory clinic of Beijing Xuanwu Hospital of Capital Medical University and the local community in China from May 2011 to June 2016. Written informed consent was obtained from all subjects before inclusion. This study has been registered to ClinicalTrials.gov (NCT02225964^1^).

The patients with aMCI were diagnosed on the basis of Petersen’s criteria (Petersen, 2004) and the National Institute on Aging Alzheimer’s Association criteria for aMCI due to AD (Albert et al., 2011) as follows: (a) with subjective memory complaint, preferably confirmed by an informant; (b) objective memory impairment confirmed by Mini-Mental State Examination (MMSE), Montreal Cognitive Assessment (MoCA), Auditory Verbal Learning Test (AVLT); (c) a Clinical Dementia Rating (CDR) score of 0.5; (d) did not fulfill the criteria for dementia according to the Diagnostic and Statistical Manual of Mental Disorders, fourth edition, revised (DSM-IV); and (e) hippocampal atrophy observed by structural MRI.

The inclusion criteria of SCD, based on the research criteria for SCD (Jessen et al., 2014) and described in our previous study (Sun et al., 2016), included the following: (a) self-reported persistent cognitive decline within the last 5 years, which was confirmed by an informant; (b) performance within the normal range on a Chinese version of the MMSE and the Beijing version of the MoCA (adjusted for age, sex, and education); and (c) a score of 0 on the CDR.

The NC participants were healthy volunteers who met the following conditions: (a) no subjective or objective cognitive decline concerns; (b) normal performance on neuropsychologic test scores; and (c) CDR score of 0.

Subjects were excluded if they had any of the following: (a) structural abnormalities that could impair cognitive function other than cerebrovascular lesions, such as tumor, subdural hematoma, and contusion from a previous head trauma; (b) a history of stroke, addictions, neurologic or psychiatric diseases, or treatments that would affect cognitive function; (c) focal neurologic signs or symptoms (e.g., paralysis, sensory disturbances, dysarthria, gait disorder, and Babinski sign); (d) depression (a score of >7 on the Hamilton depression rating scale); (e) large-vessel disease (e.g., cortical and/or subcortical infarcts and watershed infarcts); (f) and diseases with WM lesions (e.g., normal pressure hydrocephalus and multiple sclerosis). The diagnosis was performed by three neurologists who had between 8 and 28 years of experience. Clinical and demographic data for all 138 participants are shown in Table 1.

**TABLE 1:** Clinical and demographic of amnestic mild cognitive impairment (aMCI), subjective cognitive decline (SCD), and normal controls (NC).

	**NC**	**SCD**	**aMCI**	***P* value**			
				**NC vs. SCD vs. aMCI**	**NC vs. SCD**	**NC vs. aMCI**	**SCD vs. aMCI**
*N* (M/F)	51 (18/33)	36 (15/21)	51 (22/29)	0.70^a^	0.55^a^	0.42^a^	0.89^a^
Age, years	62.22 ± 9.14	63.47 ± 8.78	64.06 ± 9.54	0.59^b^	0.52^c^	0.32^c^	0.77^c^
Education, years	11.23 ± 4.63	11.44 ± 4.59	9.55 ± 4.14	0.08^b^	0.84^c^	0.06^c^	0.05^c^
AVLT: immediate recall	8.83 ± 1.92	8.14 ± 1.87	5.70 ± 1.53	<0.0001^b^	0.10^c^	<0.0001^c^	<0.0001^c^
AVLT: delayed recall	10.04 ± 3.00	8.56 ± 2.79	3.32 ± 2.87	<0.0001^b^	0.02^c^	<0.0001^c^	<0.0001^c^
AVLT: recognition	11.69 ± 3.23	10.89 ± 2.28	7.38 ± 4.17	<0.0001^b^	0.21^c^	<0.0001^c^	<0.0001^c^
MoCA	28.14 ± 1.99	25.74 ± 2.10	19.34 ± 4.27	<0.0001^b^	0.62^c^	<0.0001^c^	<0.0001^c^
MMSE	28.14 ± 2.01	27.53 ± 1.72	23.78 ± 3.29	<0.0001^b^	0.14^c^	<0.0001^c^	<0.0001^c^

*Plus-minus values are means ± S.D.*

*^*a*^The *P* value for gender distribution in the three groups was obtained by Chi-square test.*

*^*b*^The *P* values were obtained by an analysis of covariance.*

*^*c*^The *P* values were obtained by a two-sample *t*-test.*

*MMSE, Mini-Mental State Examination; NC, normal control; SCD, subjective cognitive decline; aMCI, amnestic mild cognitive impairment.*

### Data Acquisition

All of the participants were imaged with a 3.0-T MR imager (Magnetom Trio Tim; Siemens, Erlangen, Germany) at the Department of Radiology, Xuanwu Hospital, Capital Medical University. The T1-weighted images were acquired using a magnetization prepared rapid gradient echo sequence with the following parameters: repetition time (TR) = 1,900 ms; echo time (TE) = 2.2 ms; flip angle = 9°; acquisition matrix = 256 × 224; field of view (FOV) = 256 × 224 mm^2^; slice thickness = 1 mm; no gap; 176 sagittal slices; and average = 1. The DTI data were acquired using a single-shot EPI sequence with the following parameters: TR = 11,000 ms; TE = 98 ms; flip angle = 90°; acquisition matrix = 128 × 116; FOV = 256 × 232 mm^2^; slice thickness = 2 mm; no gap; 60 axial slices; and average = 3. Thirty non-linear diffusion weighting directions with *b* = 1,000 s/mm^2^ and one b0 image were obtained. All images were reviewed, and leukoencephalopathy and vascular comorbidity were evaluated by an experienced neuroradiologist with 18 years of experience in clinical radiology.

### Image Preprocessing

All DTI imaging data preprocessing was performed with the FDT toolbox in FSL^2^. Briefly, each diffusion-weighted image was coregistered to the b0 image for eddy current and head motion correction. Accordingly, the b-matrix was reoriented based on the transformation matrix (Leemans and Jones, 2009). For each voxel, the diffusion tensor elements (Basser et al., 1994), fractional anisotropy (FA) value and mean diffusivity (MD) were estimated (Basser and Pierpaoli, 1996).

### Network Construction

A network consists of nodes and edges. As shown in Supplementary Figure 1, the following procedures were applied to construct WM structural networks.

#### Network Node Definition

The automated anatomic labeling (AAL) template (Tzourio-Mazoyer et al., 2002) was used to parcel the brain into 90 regions of interest (Supplementary Table 1), which represent nodes in the WM structural network. The procedure was performed using SPM8 software^3^ and has been previously described (Zalesky et al., 2010; Bai et al., 2012; Cao et al., 2013). Briefly, we first coregistered individual T1-weighted images to the b0 images in DTI space. Then, we transformed the T1 images in DTI space into the ICBM152 T1 template in Montreal Neurological Institute (MNI) space. Next, the AAL template from the MNI space was warped to the DTI native space by applying the inverse transformation obtained from the previous step. We used a nearest-neighbor interpolation method to preserve discrete labeling values.

#### WM Tractography

Diffusion tensor tractography was carried out with the “fiber assignment by continuous tracking (FACT)” method (Mori et al., 1999) included in the Diffusion Toolkit software^4^. Briefly, we seeded the voxels with FA greater than 0.2 to compute all the tracts in the diffusion-tensor imaging dataset. For each voxel, eight seeds were evenly distributed. Each streamline was reconstructed starting from each seed following the main diffusion direction from voxel to voxel. The tractography was terminated if it turned at an angle greater than 45° or reached a voxel with an FA less than 0.2.

#### Network Edge Definition

Each pair of nodes was considered structurally connected if there was at least one streamline whose end points were located in the pair (Zalesky et al., 2011; Bai et al., 2012; Shu et al., 2012). Then, three weighted networks were constructed for each subject: the fiber number (FN)-weighted network, which used the fiber number between two regions as the weight of edges; the FA-weighted network, which used the mean FA of all the voxels on all the fibers between two regions as the weight of edges; and the MD-weighted network, which used the mean MD of all the voxels on all the fibers between two regions as the weight of edges. These three networks had the same topology but conveyed different biophysical properties (Wee et al., 2011). The networks provide the fiber numbers, degree of anisotropy and average diffusivity of fibers connecting a pair of regions.

The 4,005 × 3 = 12,015 edges in the three networks were extracted for each subject as features that were used to classify the NC, SCD, and aMCI.

### Feature Selection

Selecting a small subset of features with the greatest discriminative power has been shown to improve the classification performance and avoid overfitting (Dosenbach et al., 2010) because some features are irrelevant or redundant for classification. Several studies have suggested this can also speed up computation (De Martino et al., 2008; Pereira et al., 2009). Therefore, we adopted a univariate feature-filtering step in this study. Given a training dataset *x*_*k*_,*k* = 1,…,*m*, if *n*_*+*_ and *n*_*–*_ are the number of positive instances (i.e., SCD) and negative instances (i.e., NC), respectively, then the F-score of the *i*-th feature can be calculated as:

(1) $$\text{F}\,\left(\text{i}\right)=\frac{{\left({\bar{x}}_{i}^{\left(+\right)}-{\bar{x}}_{i}\right)}^{2}+{\left({\bar{x}}_{i}^{\left(-\right)}-{\bar{x}}_{i}\right)}^{2}}{\frac{1}{n_{+}-1}\,\sum_{k=1}^{n_{+}}{\left(x_{k,i}^{\left(+\right)}-{\bar{x}}_{i}^{\left(+\right)}\right)}^{2}+\frac{1}{n_{-}-1}\,\sum_{k=1}^{n_{-}}{\left(x_{k,i}^{\left(-\right)}-{\bar{x}}_{i}^{\left(-\right)}\right)}^{2}}$$

Where ${\bar{x}}_{i},{\bar{x}}_{i}^{\left(+\right)},{\bar{x}}_{i}^{\left(-\right)}$ are the average of the *i*-th feature of the whole, positive, and negative data sets, respectively; $x_{k,i}^{\left(+\right)}$ is the *i*-th feature of the *k*-th positive instance; and $x_{k,i}^{\left(-\right)}$ is the *i*-th feature of the *k*-th negative instance. The numerator indicates the variance between groups, and the denominator indicates the variance within each of the two groups. The larger the F-score is, the more likely the feature is to be more discriminative. Therefore, we used this score as a feature selection criterion.

Considering that univariate feature selection may overlook the multivariate pattern, we also used a multivariate method, lasso regression, to select features and compared the performance of these two feature selection methods. Because lasso is a penalized least squares method, it performs continuous shrinkage and automatic variable selection simultaneously. There is a hyperparameter to control the degree to norm regularization. We used a nested fivefold cross validation to obtain the optimal hyperparameter.

### Multiple-Kernel SVM

Given *n* training samples with $x_{i}=\left\{x_{i}^{\left(1\right)},\ldots ,x_{i}^{\left(M\right)}\right\}$ denoting the feature vector of the *i*-th sample (*M* = number of white matter networks, *m* = 1,…,*M* and $x_{i}^{\left(m\right)}=\left\{e\,d\,g\,e_{i}^{\left(m\right)}\,\left(1\right),\ldots ,e\,d\,g\,e_{i}^{\left(m\right)}\,\left(4005\right)\right\}$), *y*_*i*_ ∈ {−1, 1} denoting the corresponding label, the primal optimization problem of a conventional single kernel SVM is defined as

(2) $${\text{min}}_{w,b,\xi_{i}}\;\frac{1}{2}\,{||w||}^{2}+C\,\sum_{i=1}^{n}\xi_{i},$$

$$\mathrm{subject}\,\mathrm{to}\,y_{i}\times \left(W^{T}\,\phi \,\left(x_{i}\right)+b\right)\geq 1-\xi_{i}$$

$$\mathrm{and}\;\xi_{i}\geq 0,\text{for}\,i=1,\ldots ,n$$

where *w*, *C*, *ξ*_*i*_, ϕ(⋅), and *b* denote the normal vector to the hyperplane, the model parameter that determines the number of constraint violations, the distance of the *i*-th misclassified observation from its correct side of the margin, the kernel function and the bias term, respectively.

Normally, Eq. (2) is solved using its dual form with the kernel approach. The dual form is given as

(3) $${\text{max}}_{\alpha }\,\sum_{i=1}^{n}\alpha_{i}-\frac{1}{2}\,\sum_{i,j}\alpha_{i}\,\alpha_{j}\,y_{i}\,y_{j}\times k\,\left(x_{i},x_{j}\right),$$

$$\mathrm{subject}\,\mathrm{to}\,\sum_{i=1}^{n}\alpha_{i}\,y_{i}=0;$$

and⁢    0≤αi≤C,for⁢i=1,…,n

where α is the Lagrange multiplier and *k*(*x*_*i*_,*x*_*j*_) is the kernel function for training samples, *x*_*i*_ and *x*_*j*_.

To integrate the three networks, we used a multiple kernel SVM whose primal optimization problem can be defined as

(4) $${\text{min}}_{w^{\left(m\right)},b,\xi_{i}}\,\frac{1}{2}\,\sum_{m=1}^{M}\beta_{m}\,{||w^{\left(m\right)}||}^{2}+C\,\sum_{i=1}^{n}\xi_{i},$$

$$\mathrm{subject}\,\mathrm{to}\;y_{i}\times \left[\sum_{m=1}^{M}\beta_{m}\,\left({\left(w^{\left(m\right)}\right)}^{T}\,\phi^{\left(m\right)}\,\left(x_{i}^{\left(m\right)}\right)+b\right)\right]\geq 1-\xi_{i}$$

$$\mathrm{and}\;\xi_{i}\geq 0,\text{for}\,i=1,\ldots ,n$$

where β_*m*_ is the weighting factor on the *m*-th networks. Similarly, the corresponding dual form is given as

(5) $${\text{max}}_{\alpha }\,\sum_{i=1}^{n}\alpha_{i}-\frac{1}{2}\,\sum_{i,j}\alpha_{i}\,\alpha_{j}\,y_{i}\,y_{j}\,\sum_{m=1}^{M}\beta_{m}\,k^{\left(m\right)}\,\left(x_{i}^{\left(m\right)},x_{j}^{\left(m\right)}\right),$$

$$\mathrm{subject}\,\mathrm{to}\,\sum_{i=1}^{n}\alpha_{i}\,y_{i}=0;$$

and⁢    0≤αi≤C⁢for⁢i=1,…,n

where $k^{\left(m\right)}\,\left(x_{i}^{\left(m\right)},x_{j}^{\left(m\right)}\right)$ is the kernel function for the *m*-th networks.

Given a new test sample *x* = {*x*^(1)^,…,*x*^(*M*)^}, the decision function for the predicted label can be determined as

(6) $$F\,\left(X\right)=\text{sign}\,\left(\sum_{i=1}^{n}\alpha_{i}\,y_{i}\,\sum_{m=1}^{M}\beta_{m}\,k^{\left(m\right)}\,\left(x_{i}^{\left(m\right)},x^{\left(m\right)}\right)+b\right).$$

The multiple kernel SVM can be naturally embedded into the conventional single kernel SVM framework by noting $k\,\left(x_{i},x_{j}\right)=\sum_{m=1}^{M}\beta_{m}\,k^{\left(m\right)}\,\left(x_{i}^{\left(m\right)},x_{j}^{\left(m\right)}\right)$ as a mixed kernel between the multiple networks training samples *x*_*i*_ and *x*_*j*_ and $k\,\left(x_{i},x\right)=\sum_{m=1}^{M}\beta_{m}\,k^{\left(m\right)}\,\left(x_{i}^{\left(m\right)},x^{\left(m\right)}\right)$ as a mixed kernel between the multiple networks training samples between *x*_*i*_ and the test sample *x*.

### Support Vector Machine Training and Classification

**FIGURE 1:**
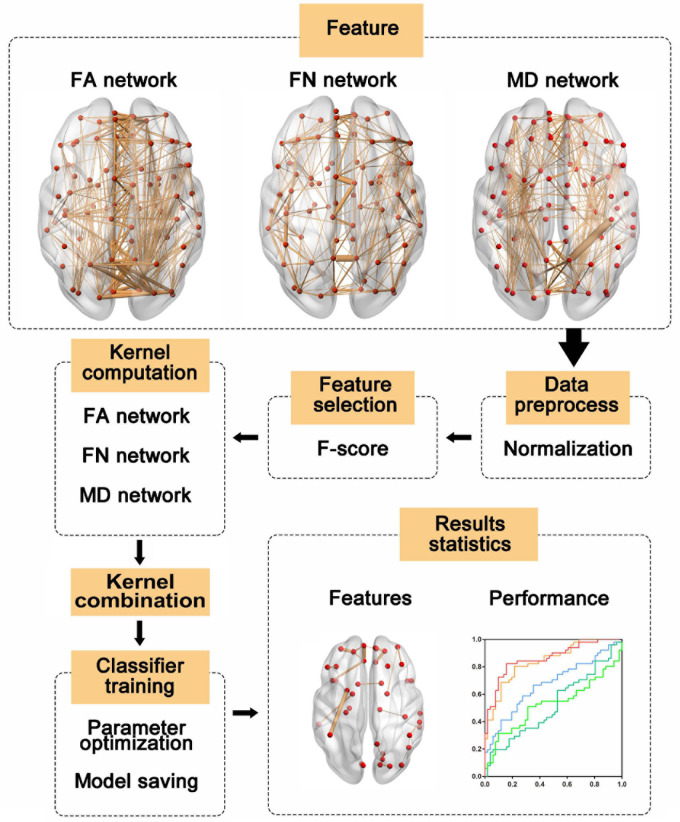
The multi-kernel support vector machine (SVM) procedure. First, features were extracted from three weighted networks and normalized with the mean value and standard deviation of the training sample. Then, features were selected according to F-score and kernel matrices were computed based on the selected features. Next, the kernel matrices were used to train the models, and the label of the test sample was predicted with trained models. Finally, we evaluated the model performances and identified the most discriminative features. FA, fractional anisotropy; FN, fiber number; MD, mean diffusivity.

The SVM classifier was trained based on the simple MKL (Rakotomamonjy et al., 2008) toolbox, which can train the weighting factors of different kernels. Due to the size limitations of the dataset, leave-one-out cross validation (LOOCV) was used to estimate the performance of the classifier. In LOOCV, each sample was considered the test sample, while the remaining samples were used to train the classifier. Before feature selection performed in training samples, the features of the test samples and training samples were normalized by using the mean value and standard deviation of the training sample. Then, the kernel matrix for each network was calculated. Finally, we trained and tested the classifier with the test sample. To obtain optimal performance, the hyperparameter C and feature number were determined by grid searching. The procedure for multi-kernel SVM training and classification is shown in Figure 1. We also applied the same pipeline to train a single-kernel SVM classifier with a single weighted network and a single-kernel SVM with multiple weighted networks. The accuracy, sensitivity and specificity were used to quantify the performance of the classifier.

(7) $$\text{Accuracy}=\frac{T\,P+T\,N}{T\,P+F\,N+T\,N+F\,P}$$

(8) $$\text{Sensitivity}=\frac{T\,P}{T\,P+F\,N}$$

(9) $$\text{Specificity}=\frac{T\,N}{T\,N+F\,P}$$

where *TP*, *FN*, *TN*, and *FP* denote the number of positive instances correctly predicted, the number of positive instances classified as negative instances, the number of negative instances correctly predicted and the number of negative instances classified as positive instances, respectively.

### Identification of the Most Discriminative Features

The essence of classification is determining a separating hyperplane. Previous studies have shown that the coefficients of the separating hyperplane quantify the power of discriminative feature information (Mourao-Miranda et al., 2005). The absolute value of the coefficients was multiplied by the weight of the corresponding network as feature weights. The higher the feature weights were, the more discriminative the corresponding features were. In every fold of LOOCV, the selected features differed slightly from fold to fold. Therefore, only the features that appeared in every fold of LOOCV were considered the most discriminative features. Each feature weight was averaged from all folds of LOOCV. To further explore which edge is most discriminative, the weights of each edge were obtained by summing the corresponding edge weights of different networks. We also determined the total weights of each network by computing the sum of feature weights from the corresponding network.

## Results

### Classification Based on Multi-Weight Networks

**FIGURE 2:**
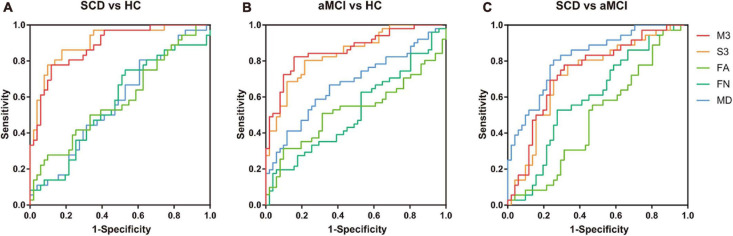
Receiver operating characteristics (ROC) of three pairs of classifications. **(A)** ROC curves of classifying SCD and NC with different models. **(B)** ROC curves of classifying aMCI and NC with different models. **(C)** ROC curves of classifying SCD and aMCI with different models. M3, multi-kernel with FA, FN, and MD weighted network; S3, single-kernel with FA, FN, and MD weighted network. ROC, receiver operating characteristic; NCs, normal controls; SCD, subjective cognitive decline; aMCI, amnestic mild cognitive impairment; FA, fractional anisotropy; FN, fiber number; MD, mean diffusivity.

A LOOCV was used to estimate the generalizability of the classifier. As shown in Table 2, the models using F-score outperformed those using lasso, so the subsequent analyses were based on the results from F-score. The proposed multiple kernel SVM-based multi-weight network approach achieved a classification accuracy of 83.9%, with a sensitivity of 77.8% and a specificity of 88.2% in the discrimination between NC subjects and SCD subjects. For the classification between NC subjects and aMCI subjects, the proposed method yielded an accuracy of 83.3%, with a specificity of 84.3% and a sensitivity of 82.4%. The task of discriminating between aMCI subjects and SCD subjects was more difficult than the other classifications, and the proposed method achieved an accuracy of 72.4%, with a specificity of 69.4% and a sensitivity of 74.5%. The three pairs of classification performances using single and multi-weight networks are summarized in Table 2. The receiver operating characteristics (ROC) curves for all compared methods in the three pairs of classifications are shown in Figure 2. Overall, multiple kernel SVM-based multi-weight networks approach achieved relatively high performance in three pair of classifications, while other methods were not robust across different tasks.

**TABLE 2:** Classification performance of the single network and multi-network methods.

**Feature selection**	**Method**	**NC and SCD**			**NC and aMCI**			**SCD and aMCI**		
		**Acc (%)**	**Sen (%)**	**Spe (%)**	**Acc (%)**	**Sen (%)**	**Spe (%)**	**Acc (%)**	**Sen (%)**	**Spe (%)**
F-score	Multi-kernel (FA, FN, and MD)	83.9	**77.8**	88.2	**83.3**	**82.4**	**84.3**	72.4	69.4	74.5
	Single-kernel (FA, FN, and MD)	**85.1**	77.8	**90.2**	77.5	78.4	76.5	71.3	69.4	72.6
	FA	58.6	47.2	66.7	54.9	56.9	52.9	52.9	50.0	54.9
	FN	56.3	44.4	64.7	53.9	60.8	47.1	64.4	52.8	72.6
	MD	56.3	47.2	62.7	75.5	56.9	74.5	**73.6**	69.4	**76.5**
Lasso	Multi-kernel (FA, FN, and MD)	73.6	72.2	74.5	75.5	78.4	72.5	62.1	60.8	63.9
	Single-kernel (FA, FN, and MD)	75.9	77.8	74.5	71.6	72.5	70.6	69.0	**70.6**	66.7
	FA	49.4	63.9	39.2	51.0	45.1	56.9	39.1	49.0	25.0
	FN	56.3	55.6	56.9	52.9	51.0	54.9	54.0	68.6	33.3
	MD	51.7	55.6	49.0	60.8	68.6	52.9	62.1	70.6	50.0

*Acc, accuracy; Sen, sensitivity; Spe, specificity; NC, normal control; SCD, subjective cognitive decline; aMCI, amnestic mild cognitive impairment; FA, fractional anisotropy; FN, fiber number; MD, mean diffusivity. The bold values represent the best performance.*

### Effect of Constraint Parameter C in Linear Kernel and Nonlinear Kernel Function

**FIGURE 3:**
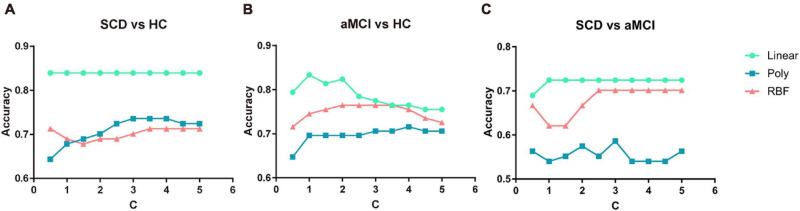
Classification accuracy of three pairs of classifications under different parameter C value and different kernel functions. **(A)** The performance of classifying SCD and NC under different C values. **(B)** The performance of classifying aMCI and NC under different C values. **(C)** The performance of classifying SCD and aMCI under different C values. NC, normal control; SCD, subjective cognitive decline; aMCI, amnestic mild cognitive impairment.

To investigate the influence of different constraint parameter C on the classification performance, the feature number was fixed, and the constraint parameter C was varied from 0.5 to 5 in steps of 0.5. The three pairs of classification accuracies with multi-kernel SVMs using different kernel functions and the corresponding C value are shown in Figure 3.

For every value of C, the multi-kernel SVM with a linear kernel yielded the highest accuracy compared to the multi-kernel SVM with a polynomial kernel and radial basis function (RBF) kernel. The multi-kernel SVM with a linear kernel was the most robust to C. The performance of the proposed method was nearly unchanged under the variation of constraint parameter C.

### Effect of Number of Features in the Linear Kernel and Nonlinear Kernel Function

**FIGURE 4:**
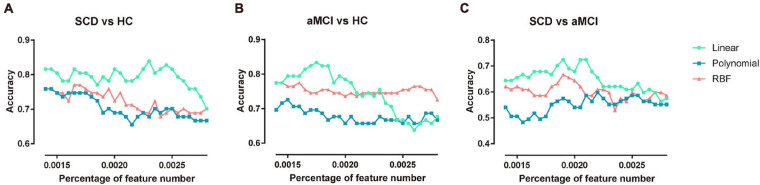
Classification accuracy of three pairs of classifications under different feature numbers and different kernel functions. **(A)** The performance of classifying SCD patients and NC under different selected feature numbers. **(B)** The performance of classifying aMCI and NC under different selected feature numbers. **(C)** The performance of classifying SCD and aMCI under different selected feature numbers. NC, normal control; SCD, subjective cognitive decline; aMCI, amnestic mild cognitive impairment.

In the proposed framework, the F-score was applied to select a subset of features with the most discriminative power. The features with higher F-scores were input to train the model. Therefore, the percentage of features to be selected is determined by the predefined value. In this subsection, to explore the robustness of the multi-kernel SVM, the constraint parameter C was fixed as 1, and the percentage of feature numbers was varied from 0.0014 to 0.0028 in steps of 0.00005. The three pairs of classification accuracies with multi-kernel SVMs using different kernel functions and the corresponding percentage of selected features are summarized in Figure 4.

The multi-kernel SVM with a linear kernel yielded the highest accuracy compared to the multi-kernel SVM with polynomial and RBF kernels at the corresponding percentage of feature numbers. For a higher percentage of feature numbers, classification accuracy decreased because the larger amount of features included some redundant and confounding features.

### The Most Discriminant Regions

**FIGURE 5:**
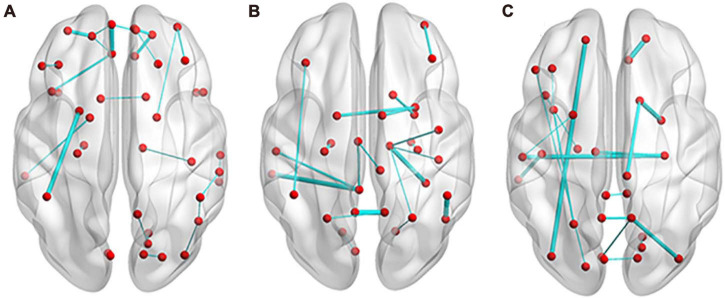
Edges with most discrimination power. **(A)** In the classification of SCD and NC, 27 edges appeared in every fold of leave-one-out cross validation (LOOCV). **(B)** For the discrimination of aMCI and NC, 20 edges appeared in every fold of LOOCV. **(C)** Between SCD and aMCI, 18 edges appeared in every fold of LOOCV. The thickness of the edges represents the weight. NC, normal control; SCD, subjective cognitive decline; aMCI, amnestic mild cognitive impairment.

In the classification of SCD and NC, 35 features (14 features from the MD network, 14 features from the FA network, and 7 features from the FN network) appeared in every fold of LOOCV (Supplementary Table 2). As shown in Figure 5A, the edges with great relative classification power included the connection between the left medial orbital of the superior frontal gyrus (ORBsupmed) and left rectus (REC), the connection between the left putamen (PUT) and left inferior partial lobe (IPL), the connection between the left orbital of the middle frontal gyrus (ORBmid) and left orbital of the superior frontal gyrus (ORBsup), the connection between the right ORBsup and right REC, and the connection between the right ORBsupmed and right ORBsup. The total weights of the MD network, FA network, and FN network are 183.75, 145.84, and 47.58, respectively.

For the discrimination of aMCI and NC, 28 features (15 features from the MD network, nine features from the FA network, and 14 features from the FN network) appeared in every fold of LOOCV (Supplementary Table 3). As shown in Figure 5B, the edges with great relative classification power included the connection between the left precuneus (PCUN) and right PCUN, the connection between the right fusiform gyrus (FFG) and right thalamus (THA), the connection between the left middle temporal gyrus (MTG) and left posterior cingulum gyrus (PCG), and the connection between the right PUT and left PUT. The total weights of the MD network, FA network and FN network are 479.90, 137.37, and 115.09, respectively.

Between SCD and aMCI, 27 features (nine features from the MD network, 10 features from the FA network, and eight features from the FN network) appeared in every fold of LOOCV (Supplementary Table 4). As shown in Figure 5C, the edges with great relative classification power included the connection between the right ORBsup and right REC, the connection between the right amygdala (AMYG) and right caudate (CAU), the connection between the left ORBsup and left inferior occipital gyrus (IOG), the connection between the right hippocampus (HIP) and left superior temporal gyrus (STG), and the connection between the right middle occipital gyrus (MOG) and right PCUN. The total weights of the MD network, FA network, and FN network are 183.43, 109.39, and 85.47, respectively.

## Discussion

In the current study, we established an efficient classification framework using a multi-kernel SVM based on multi-weight networks, enabling us to distinguish SCD and aMCI patients from NCs with accuracies of 83.9 and 83.3%, respectively. Previous studies have reported accuracy levels ranging from 59.2 to 88.9% for DTI data in the classification of aMCI and NC (Wee et al., 2012; Dyrba et al., 2015; Prasad et al., 2015). In our classification of SCD and NC, an area under the curve (AUC) value of 0.89 was obtained, with an accuracy of 83.9%, sensitivity of 77.8%, and specificity of 88.2%. Considering the relatively subtle alternations in the SCD population, our methods indicate its excellent diagnostic power. Moreover, our proposed classification framework herein relies on a simpler DTI scanning protocol and thus requires less image acquisition effort. This makes the approach more economical and clinically feasible.

In the classification of patients and NCs, the classification accuracy of the multi-kernel approach and direct data fusion method was significantly higher than that of any single weight network approach. The limited information provided by a single WM-weighted network may not be enough for distinguishing SCD and aMCI patients from NCs, as indicated by the much smaller AUC values. Although the multi-kernel approach resulted in slightly inferior accuracy than the direct data fusion method in classification between SCD and NC and the MD-weighted network in discrimination of aMCI and SCD, it was a great overall performer for the three pairs of classifications.

Direct data fusion method suffers from a major pitfall that it may produce models that effectively ignore the modalities that have less features while multi-kernel method does not have this problem because it treats all modalities as equivalent no matter how many features they have (Rathore et al., 2017). In this study, all modalities had the same number of features. So it seems that multi-kernel method did not have clear advantages in the classification between SCD and NC.

In the classification between SCD and aMCI, the results showed MD-weighted network outperformed other models, even the models from multi-weight networks. In addition, MD- and FA-weighted network almost equally contributed to the classification between SCD and NC, while the classification between aMCI and NC was mainly determined by MD-weighted network. It’s probably because that MD is more sensitive than FA and FN in revealing early pathological process (Wang et al., 2020). Hence, FA- and FN- weighted network were so redundant for the classification between SCD and aMCI that adding them into the classification lowered the performance.

Comparing the performance between the classification between NCs and SCD patients and the classification between NCs and aMCI patients, we found that the model classifying SCD patients from NCs had slightly higher accuracy as it had comparatively higher specificity. However, the model classifying aMCI patients from NCs had higher sensitivity. These evidences means the classifier between aMCI and NC is more sensitive to patients than the classifier between SCD and NC. It’s probably because SCD patients’ WM alterations are subtle and intermediate between those in aMCI and NC (Brueggen et al., 2019). So the model classifying SCD patients from NCs tended to label test sample as NC while the model classifying aMCI patients from NCs can better identify patients.

Compared with NC, WM structural network patterns of patients with SCD and aMCI were significantly altered. The most discriminant regions selected for accurate detection of individuals with SCD were from MD- and FA-weighted networks, which include connections among the prefrontal cortex, orbitofrontal cortex, parietal lobe and temporal regions. Some regions like ORBsupmed and hippocampus locate in the default mode network, which are most vulnerable by amyloid (Wang et al., 2020). This indicates that the early deposition of amyloid may impaired the WM connectivities in these regions. From the view of graph theory, we have previously observed less network efficiency and connection strength of the brain structural connectome among these regions in the SCD group (Shu et al., 2018). Moreover, this impaired capacity of information transfer may derive from WM microstructure abnormalities with decreased FA and increased MD patterns observed in SCD subjects, which were demonstrated by previous studies (Selnes et al., 2012; Li et al., 2016). Between aMCI patients and NCs, most discriminative features were from the MD-weighted network and were distributed across parietal, temporal, and frontal lobes, which is largely in line with previous studies (Wee et al., 2011; Selnes et al., 2012; Shu et al., 2012). We can see that there was a difference in the distribution of selected features between the two models. In the classification between SCD and NC, the major features were the connections in frontal lobe while the features mostly located in posterior parietal lobe like precuneus and subcortical nuclei such as hippocampus and thalamus when classifying aMCI and NC. This difference may indicate a pathological development of AD that initial impairment in frontal lobe diffuses to the parietal lobe and subcortical nuclei, which is consistent with a preview study (Yan et al., 2019).

In addition, we investigated the effect of the constraint parameter C and selected feature number for classification performance. The multi-kernel SVM with a linear kernel was found to be most stable and robust to constraint parameter C and feature number compared with the multi-kernel SVM with a polynomial kernel and RBF kernel. These results suggest that the dataset that we acquired and analyzed in this study is more linearly separable than nonlinearly separable. This may be contradictory to reports in a previous study (Wee et al., 2011, 2012). The discrepancy might be due to methodological differences in parameter selection and image analysis. The performance of the model decreased with an increase in the selected feature number when the feature number exceeded a value, which was nearly consistent among models with different kernel functions. This suggests there were some irrelevant and redundant features that had adverse impact on model performance. Therefore, it is important to perform feature selection before training models.

There are some limitations of our study that should be considered. One limitation of our current study is the relatively limited sample size compared to the dimensionality of the connectivity measurements. Although the LOOCV accuracy obtained may be optimistic, the restricted sample size did not allow us to explore other cross-validation techniques since the nonlinear SVM classifier used might be undertrained. Second, we only identified classification performance in patients with SCD, and longitudinal follow-up studies of the same study population are needed to further confirm our results. Third, the diagnosis of SCD and aMCI were not confirmed by amyloid PET. Forth, the generalizability of the findings is unclear without independent validation dataset. Finally, we only studied WM structural networks. In future studies, whether a combination of multimodal imaging (i.e., structural and/or functional MR imaging) and CSF biomarkers and genetic data provides additional diagnostic accuracy for the SCD population should be further clarified.

In conclusion, a multiple-kernel SVM based on a multi-weight network approach has been proposed to describe the complex WM connectivity patterns for automatically identifying individuals with SCD and aMCI from NCs. The promising results indicate that the proposed classification framework can facilitate and possibly improve individualized clinical diagnosis of alterations in brain structure associated with SCD.

## Data Availability Statement

The raw data supporting the conclusions of this article will be made available by the authors, without undue reservation.

## Ethics Statement

The studies involving human participants were reviewed and approved by Beijing Xuanwu Hospital of Capital Medical University. The patients/participants provided their written informed consent to participate in this study.

## Author Contributions

YH and NS conceived, designed, revised, and finalized the manuscript. WH and XuL contributed equally to perform data analyses and wrote the manuscript. XiL and GK revised and provided critical input to the manuscript. All authors read and approved the final manuscript.

## Conflict of Interest

The authors declare that the research was conducted in the absence of any commercial or financial relationships that could be construed as a potential conflict of interest. The reviewer LW declared a shared affiliation, with no collaboration, with several of the authors XuL and YH to the handling editor at the time of the review.

## Publisher’s Note

All claims expressed in this article are solely those of the authors and do not necessarily represent those of their affiliated organizations, or those of the publisher, the editors and the reviewers. Any product that may be evaluated in this article, or claim that may be made by its manufacturer, is not guaranteed or endorsed by the publisher.

## Footnotes

1
https://www.clinicaltrials.gov/

2
https://fsl.fmrib.ox.ac.uk/fsl/fslwiki/

3
https://www.fil.ion.ucl.ac.uk/spm/

4
http://www.trackvis.org/dtk/
