# Supplementary material for: Combined Support Vector Machine Classifier and Brain Structural Network Features for the Individual Classification of Amnestic Mild Cognitive Impairment and Subjective Cognitive Decline Patients
Source: Front Aging Neurosci. 2021 Jul 30;13:687927. doi: 10.3389/fnagi.2021.687927 (PMC8361326; doi:10.3389/fnagi.2021.687927)
Supplement: Supplementary file 1 [file Data_Sheet_1.docx]

Supplementary materials

**Table S1. Cortical and subcortical regions of interest defined in the study.**

| **Index** | **Regions** | **Abbr.** | **Index** | **Regions** | **Abbr.** |
| --- | --- | --- | --- | --- | --- |
| (1,2) | Precental gyrus | PreCG | (47,48) | Lingual gyrus | LING |
| (3,4) | Superior frontal gyrus, dorsolateral | SFGdor | (49,50) | Superior occipital gyrus | SOG |
| (5,6) | Superior frontal gyrus, orbital part | ORBsup | (51,52) | Middle occipital gyrus | MOG |
| (7,8) | Middle frontal gyrus | MFG | (53,54) | Inferior occipital gyrus | IOG |
| (9, 10) | Middle frontal gyrus, orbital part | ORBmid | (55,56) | Fusiform gyrus | FFG |
| (11,12) | Inferior frontal gyrus, opercular part | IFGoperc | (57,58) | Postcentral gyrus | PoCG |
| (13,14) | Inferior frontal gyrus, triangular part | IFGtriang | (59,60) | Superior parietal gyrus | SPG |
| (15,16) | Inferior frontal gyrus, orbital part | ORBinf | (61,62) | Inferior parietal, but supramarginal and angular gyri | IPL |
| (17,18) | Rolandic operculum | ROL | (63,64) | Supramarginal gyrus | SMG |
| (19,20) | Supplementary motor area | SMA | (65,66) | Angular gyrus | ANG |
| (21,22) | Olfactory cortex | OLF | (67,68) | Precuneus | PCUN |
| (23,24) | Superior frontal gyrus, medial | SFGmed | (69,70) | Paracentral lobule | PCL |
| (25,26) | Superior frontal gyrus, medial orbital | ORBsupmed | (71,72) | Caudate nucleus | CAU |
| (27,28) | Gyrus rectus | REC | (73,74) | Lenticular nucleus, putamen | PUT |
| (29,30) | Insula | INS | (75,76) | Lenticular nucleus, pallidum | PAL |
| (31,32) | Anterior cingulate and paracingulate gyri | ACG | (77,78) | Thalamus | THA |
| (33,34) | Median cingulate and paracingulate gyri | DCG | (79,80) | Heschl gyrus | HES |
| (35,36) | Posterior cingulate gyrus | PCG | (81,82) | Superior temporal gyrus | STG |
| (37,38) | Hippocampus | HIP | (83,84) | Temporal pole: superior temporal gyrus | TPOsup |
| (39,40) | Parahippocampal gyrus | PHG | (85,86) | Middle temporal gyrus | MTG |
| (41,42) | Amygdala | AMYG | (87,88) | Temporal pole: middle temporal gyrus | TPOmid |
| (43,44) | Calcarine fissure and surrounding cortex | CAL | (89,90) | Inferior temporal gyrus | ITG |
| (45,46) | Cuneus | CUN |  |  |  |

Note: The regions are listed in terms of a prior template of an AAL-atlas (Tzourio-Mazoyer et al., 2002).

Table S2. The whole features of classification for SCD and NC.

| Edges | Networks | Weights | Edges | Networks | Weights |
| --- | --- | --- | --- | --- | --- |
| REC.L←→ORBsupmed.L | MD | 27.8 | CAU.R←→CAU.L | FN | 9.2 |
| ORBsupmed.R←→ORBsup.R | MD | 26.1 | ORBmid.L←→ORBsup.L | FN | 9.1 |
| PUT.L←→IPL.L | MD | 25.9 | MTG.L←→PAL.L | FA | 9.0 |
| REC.L←→ORBsupmed.L | FA | 21.7 | SMG.R←→IPL.R | MD | 8.8 |
| TPOmid.R←→TPOsup.R | MD | 20.7 | SOG.R←→CUN.R | FA | 8.7 |
| PUT.L←→IPL.L | FA | 18.9 | TPOmid.L←→TPOsup.L | FN | 8.6 |
| REC.R←→ORBsup.R | FA | 16.2 | CUN.L←→CAL.L | FN | 7.2 |
| ORBmid.L←→ORBsup.L | FA | 16.1 | MTG.L←→PAL.L | MD | 7.1 |
| ORBmid.R←→MFG.R | MD | 15.9 | PAL.R←→ORBmid.R | FA | 4.5 |
| REC.R←→ORBsup.R | MD | 14.6 | THA.R←→PoCG.R | FA | 3.6 |
| TPOsup.L←→REC.L | MD | 12.4 | PCUN.R←→CAL.R | FN | 2.3 |
| ORBsupmed.L←→ORBsup.L | FA | 12.2 | ORBsupmed.L←→ORBsup.R | FA | 1.7 |
| MTG.R←→STG.R | MD | 11.7 | TPOmid.R←→TPOsup.R | FA | 0.9 |
| TPOsup.L←→REC.L | FA | 11.2 | ANG.R←→IPL.R | MD | 0.9 |
| ORBinf.L←→IFGtriang.L | FA | 10.9 | SFGmed.R←→SFGdor.R | MD | 0.7 |
| ORBmid.L←→ORBsup.L | MD | 10.6 | ANG.R←→MOG.R | MD | 0.7 |
| LING.R←→CAL.R | FN | 10.5 | PHG.L←→HIP.L | FN | 0.6 |
| REC.L←→ORBsup.L | FA | 10.4 |  |  |  |

Table S3. The whole features of classification for aMCI and NC.

| Edges | Networks | Weights | Edges | Networks | Weights |
| --- | --- | --- | --- | --- | --- |
| ANG.R←→IPL.R | MD | 74.5 | THA.R←→FFG.R | FA | 25.1 |
| MTG.L←→PCG.L | MD | 69.0 | PCUN.R←→PCUN.L | MD | 245.0 |
| PAL.L←→PUT.R | MD | 49.0 | PCL.R←→DCG.L | FA | 21.4 |
| THA.R←→FFG.R | MD | 44.3 | THA.R←→PoCG.R | MD | 13.5 |
| PHG.L←→HIP.L | FN | 43.6 | PCUN.R←→SPG.L | MD | 12.6 |
| PUT.R←→SMA.R | MD | 43.1 | SPG.R←→LING.R | FA | 7.3 |
| PCUN.R←→PCUN.L | FA | 40.9 | LING.L←→CAL.L | MD | 6.3 |
| CAU.R←→AMYG.R | FN | 39.5 | PCUN.R←→SPG.L | FA | 5.1 |
| ORBmid.R←→MFG.R | MD | 39.2 | THA.R←→SPG.R | FA | 5.0 |
| STG.L←→PCG.L | MD | 37.5 | 'THA.R←→SPG.R | MD | 4.5 |
| PCG.L←→DCG.L | MD | 30.3 | SPG.R←→LING.R | MD | 4.1 |
| THA.R←→PreCG.R | FA | 28.7 | PCUN.R←→PCUN.L | FN | 3.8 |
| PHG.R←→HIP.R | FN | 28.2 | THA.R←→PoCG.R | FA | 3.5 |
| IPL.L←→ORBinf.L | MD | 27.3 | IPL.L←→ORBinf.L | FA | 0.6 |

Table S4. The whole features of classification for aMCI and SCD.

| Edges | Networks | Weights | Edges | Networks | Weights |
| --- | --- | --- | --- | --- | --- |
| REC.R←→ORBsup.R | MD | 44.3 | PCUN.R←→MOG.R | MD | 12.6 |
| CAU.R←→AMYG.R | FN | 41.9 | PCUN.R←→CAL.L | FN | 10.2 |
| STG.L←→HIP.R | MD | 23.1 | TPOsup.L←→PHG.L | FA | 10.0 |
| IOG.L←→ORBsup.L | MD | 22.1 | LING.R←→CAL.R | FN | 7.30 |
| PCUN.R←→MOG.R | FA | 22.1 | INS.L←→ORBinf.L | FA | 5.70 |
| THA.L←→HIP.R | MD | 19.3 | STG.L←→PUT.L | FA | 5.0 |
| CAU.R←→PCL.R | MD | 18.8 | PCG.R←→PCG.L | FN | 4.3 |
| IOG.L←→ORBsup.L | FA | 18.8 | CUN.R←→CUN.L | FN | 4.2 |
| MTG.L←→HES.L | MD | 18.8 | SOG.L←→IFGtriang.L | MD | 4.0 |
| PCUN.R←→PCUN.L | MD | 17.0 | STG.L←→PUT.L | MD | 3.5 |
| STG.L←→HIP.R | FA | 15.6 | SOG.L←→IFGtriang.L | FA | 3.1 |
| SPG.L←→INS.L | FN | 15.0 | PCUN.R←→PCUN.L | FN | 2.5 |
| MTG.L←→HES.L | FA | 14.4 | THA.L←→HIP.R | FA | 1.8 |
| CAU.R←→PCL.R | FA | 13.0 |  |  |  |


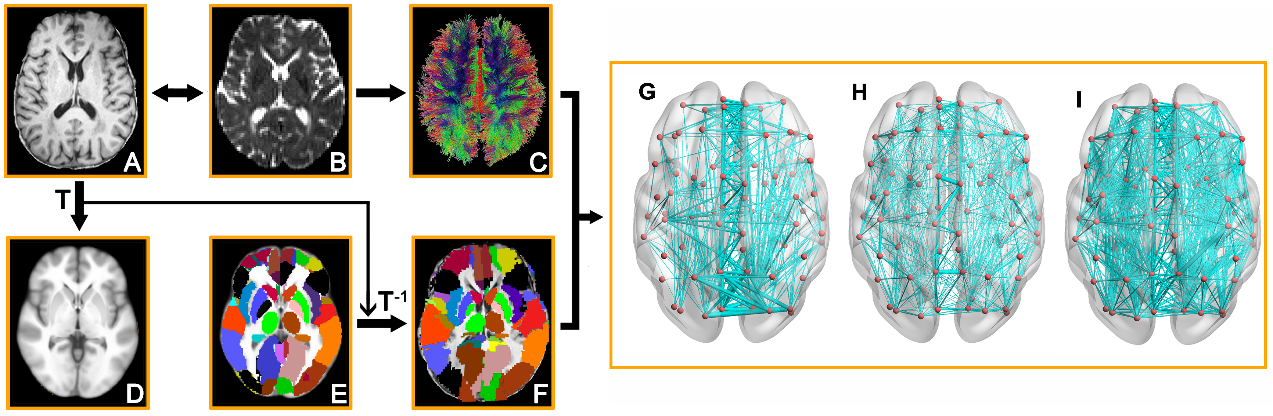


Fig. S1. The procedure of constructing whiter matter networks. (1) Coregistered T1-weighted image (A) to b0 image (B) for each subject. Nonlinearly registered the individual T1-weighted image in DTI space to the ICBM152 T1 template in MNI space (D), resulting in a nonlinear transformation (T). (3) Applied the inverse transformation (T^-1^) to the AAL template in the MNI space (E), resulting in subject-specific parcellation in the DTI native space (F). All registrations were implemented in the SPM8 package. (4) Whole-brain WM fibers (C) were constructed with deterministic tractography in the Diffusion Toolkit. (5) The FN networks of each subject (G) were created by computing the number of streamlines that connected each pair of brain regions; the FA networks (H) and MD networks (I) of each subject were created by computing the averages of on-fiber FA and MD.

DTI = Diffusion tensor image; ICBM = The McConnell Brain Imaging Centre; MNI = Montreal Neurological Institute; AAL = Automated anatomical labeling; SPM = Statistical parameter mapping; WM = Whiter matter; FN = Fiber number; FA = Fractional anisotropy; MD = Mean diffusivity.
